# The Wheat E Subunit of V-Type H^+^-ATPase Is Involved in the Plant Response to Osmotic Stress

**DOI:** 10.3390/ijms150916196

**Published:** 2014-09-12

**Authors:** Xiao-Hong Zhang, Bo Li, Yin-Gang Hu, Liang Chen, Dong-Hong Min

**Affiliations:** 1 State Key Laboratory of Crop Stress Biology for Arid Areas and College of Life Sciences, Northwest A&F University, Yangling 712100, China; E-Mail: zhxh2493@126.com; 2 State Key Laboratory of Crop Stress Biology for Arid Areas and College of Agronomy, Northwest A&F University, Yangling 712100, China; E-Mails: libo708@126.com (B.L.); huyingang@126.com (Y.-G.H.); chenliang9117@nwafu.edu.cn (L.C.)

**Keywords:** *Triticum aestivum* L., V-type H^+^-ATPase, E subunit, inducible mechanism, osmotic stress

## Abstract

The vacuolar type H^+^-ATPase (V-type H^+^-ATPase) plays important roles in establishing an electrochemical H^+^-gradient across tonoplast, energizing Na^+^ sequestration into the central vacuole, and enhancing salt stress tolerance in plants. In this paper, a putative E subunit of the V-type H^+^-ATPase gene, *W36* was isolated from stress-induced wheat *de novo* transcriptome sequencing combining with 5'-RACE and RT-PCR methods. The full-length of *W36* gene was 1097 bp, which contained a 681 bp open reading frame (ORF) and encoded 227 amino acids. Southern blot analysis indicated that *W36* was a single-copy gene. The quantitative real-time PCR (qRT-PCR) analysis revealed that the expression level of *W36* could be upregulated by drought, cold, salt, and exogenous ABA treatment. A subcellular localization assay showed that the *W36* protein accumulated in the cytoplasm. Isolation of the *W36* promoter revealed some *cis*-acting elements responding to abiotic stresses. Transgenic *Arabidopsis* plants overexpressing *W36* were enhanced salt and mannitol tolerance. These results indicate that *W36* is involved in the plant response to osmotic stress.

## 1. Introduction

Salt stress is one of the main environmental factors that cause osmotic stress and reduction in plant growth and crop productivity [1,2]. Salt stress can destroy plant membrane and make numerous Na^+^ flood into cell, and finally break up the intrinsical electric balance *in vivo* [3]. To ensure the process of photosynthesis and other important metabolisms, plants need to maintain the balance of low Na^+^ level and high K^+^, Ca^2+^, and Mg^2+^ level in cytoplasm. Plants have three major methods to remit Na^+^ infections, including restricting Na^+^ absorption, expediting Na^+^ exocytosis, and energizing Na^+^ segmentation in the vacuole [4,5]. ATPases are a class of enzymes that play crucial roles in ion transportation and plant salt resistant response. Plasma membrane ATPase and vacuolar ATPase and pyrophosphatase (PPase) are main proton pumps, which provide energy for ion transportion across plasma membrane and tonoplast, respectively. While membrane Na^+^/H^+^ antiporters could take advantage of the proton gradient formed by these pumps to exchange Na^+^ for H^+^, many evidences suggest that tonoplast Na^+^/H^+^ antiporter which drives Na^+^ from cytosol into vacuole play a major role in Na^+^ compartmentalization in plant leaves [6,7]. Therefore, under high salt condition plants can use the power energized by an electrochemical H^+^-gradient generated by primary-active H^+^ pumps located at the tonoplast, such as vacuolar type H^+^-ATPase (V-type H^+^-ATPase or V-H^+^-ATPase) and V-type H^+^-PPase to active secondary transport of Na^+^ from the cytosol into the vacuole via tonoplast Na^+^/H^+^ antiporter to eliminate Na^+^ toxicity, and consequently enhance salt resistance [8,9].

ATPases can catalyze the degradation of adenosine triphosphate (ATP) into adenosine diphosphate (ADP) and a free phosphate ion. There are three types of ATPases on plant cell membrane [10]. The first P-type ATPases located on plasma membrane is a kind of phosphorylated cation pump driven by ATP. The P-type ATPase activity is controlled by many factors, such as hormones, calcium, light, and environmental stresses [8,9,11,12,13]. For example, in *Arabidopsis*, P-type H^+^-ATPase are encoded by a 12-member gene family (*AHA1* to *AHA12*) [14,15]. Mutations in the *AHA1* produce a constitutive protein activity and reduce sensitivity to ABA-mediated stomatal closure [16]. Additionally, overexpression of an activated P-type H^+^-ATPase enhanced plant salt tolerance [17]. The second V-type ATPases located on tonoplast basically use energy produced by ATP hydrolysis process and transfer protons from cytoplasmic into vacuole to make vacuole acidification. Among of all, V-type H^+^-ATPases account for about 6%–8% of the total tonoplast proteins and even reach to 30%. The molecular weight of V-H^+^-ATPases is approximately 400–650 kD, which are composed of 7–10 subunits and divided into hydrophilic V1 subunit group (made up of 8 kinds of subunits (A-H)) and hydrophobic V0 (made up of 5–6 kinds of subunits (a, d, c, c', c'', e)). The optimal pH of this enzyme is about 7.2. It can be activated by anions, for instance Cl^−^. V-type H^+^-ATPases not only maintain the dynamic balance of cytoplasm ion and cell metabolism as a kind of “dominate enzyme”, but also respond to environmental factors through appropriately changing subunits expression and modulating enzyme activity. The structure of V-type H^+^-ATPases appears to be conserved across eukaryotes [18]. However, most plant V-type H^+^-ATPases subunits are encoded by small multigene families, which have been detected in many plant genomes [10,19]. In *Arabidopsis*, V1 subunits B, E and G are each encoded by three different VHA genes, and multiple isoforms exist for each of the five V0 subunits [20]. *Arabidopsis* VHA-c1 and c3 subunit isoforms have been knocked down by RNAi, each resulting in reduced root length and decreased tolerance to moderate salt stress [21]. The presence of multigene families suggests that genes for V-type H^+^-ATPases subunits may respond to specific developmental or environmental cues, which allow each subunit to be amplified or suppressed as required [9]. The third F-type ATPases is located on inner membrane of mitochondrial and thylakoid membrane of chloroplasts. It is mainly coupled with H^+^ transmembrane transshipment and participates in ATP synthesis process. Both F- and V-type H^+^-ATPases are composed of multiple subunit complexes. Homology sequence analyses of the catalytic and noncatalytic subunits of the F- and V-type H^+^-ATPases have suggested that these genes are derived from a common ancestral gene [22].

Wheat is an important food crop and occupies a momentous position in the national economy. Salt stress caused by soil salt and alkali seriously affects the yield and quality of wheat. In this study *W36* (ABC70183.1), a V-type H^+^-ATPase subunit E gene, was isolated from stress-induced *de novo* transcriptome sequencing of wheat. Molecular characteristics and functions of *W36* were analyzed to clarify the molecular mechanism of V-type H^+^-ATPase, which will provide theory basis for further realizing stress physiology process and completing signal-transporting systems in plants.

## 2. Results

### 2.1. cDNA Isolation and Homology Analysis

A 833 bp cDNA fragment named *W36* was isolated by analysis of the *de novo* transcriptome sequencing. Combining with 5'-RACE and RT-PCR methods, *W36* full-length sequence was obtained. The full-length of *W36* cDNA was 1097 bp, which contained a 681 bp open reading frame (ORF) and encoded 227 amino acids. The 5'-non-coding and 3'-non-coding region was 144 and 251 bp, respectively. The predicted molecular weight and *pI* value was 19.7 kD and 6.87, respectively. A vATP-synt-E (Pfam01991, 17-220 AA) conserved domain of *W36* was discovered in GenBank which was a typical characteristic of V-type H^+^-ATPase E subunit family.

**Figure 1:**
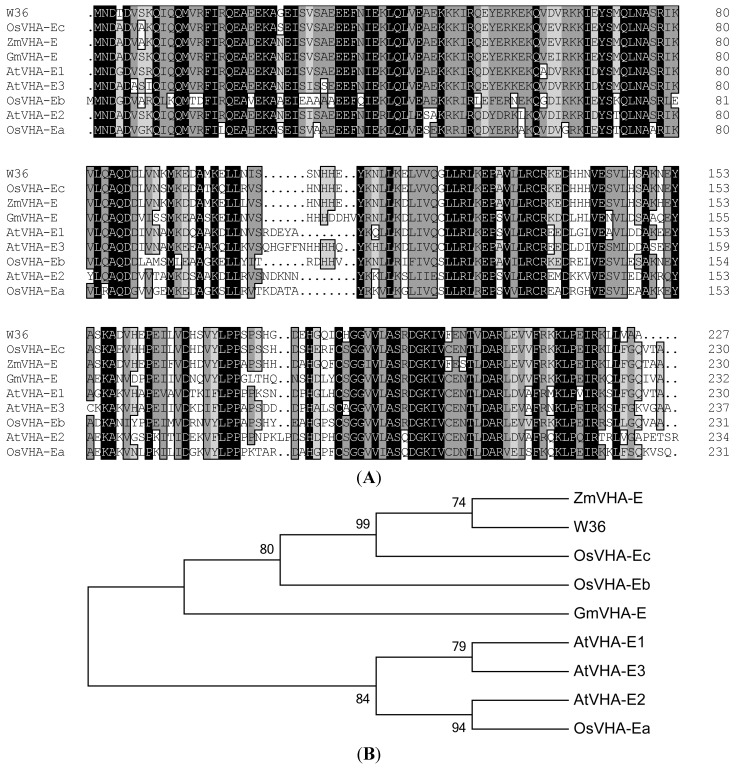
Sequence alignment and phylogenetic analysis of *W36* with other related proteins. (**A**) Sequence alignment of *W36* with other related proteins. Alignment of V-type H^+^-ATPase E subuint proteins with products of homologous genes from other plants: AtVHA-E1 (Acc. NP_192853.1), AtVHA-E2 (Acc. NP_187468.1), AtVHA-E3 (Acc. NP_176602.1), OsVHA-Ea (Acc. NP_001055857.1), OsVHA-Eb (Acc. NP_001042437.1), OsVHA-Ec (Acc. NP_001043767.1), ZmVHA-E (Acc. ACG31413.1), GmVHA-E (Acc. XP_003533059.1). Identical amino acids are in black boxes, similar amino acids are in grey boxes; (**B**) phylogenetic analysis of *W36* with other related proteins. Phylogenetic tree analysis shows that the higher similar means the more closer. The phylogenetic were generated by MEGA 5.1 using neighbor-joining method.

Figure 1A shows the alignment between the amino acid sequences of *W36* and maize VHA-E, *Glycine max* VHA-E, three rice VHA-E (OsVHA-Ea, OsVHA-Eb, and OsVHA-Ec) and three *Arabidopsis* genes, AtVHA-E1 and AtVHA-E2, and AtVHA-E3. *W36* shares an 87% identity with ZmVHA-E and showed an 86%, 75%, 73%, 63%, 72%, 65%, and 69% identity with OsVHA-Ec, GmVHA-E, AtVHA-E1, AtVHA-E2, AtVHA-E3, OsVHA-Ea, and OsVHA-Eb, respectively (Figure 1B).

### 2.2. W36 Copy Number Analysis

**Figure 2:**
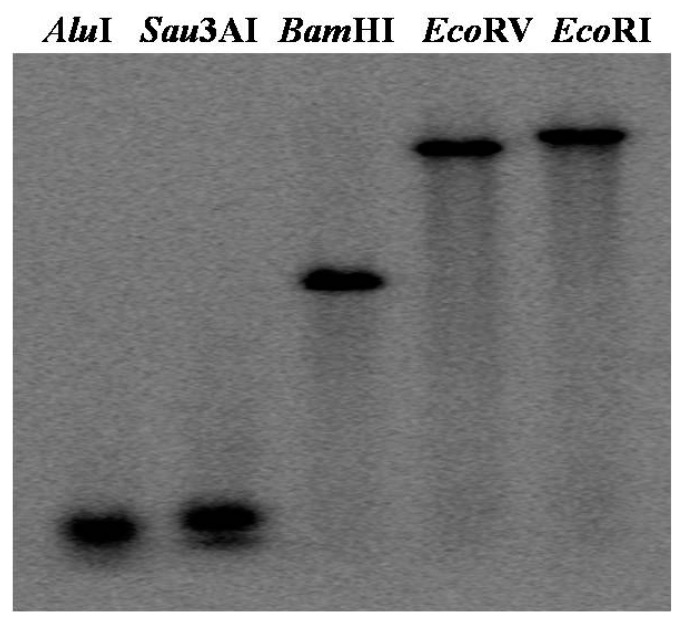
Southern blotting analysis of *W36*. Wheat genomic DNA (20 μg) from seedlings of wheat was digested by *AluI* (lane 1), *Sau3AI* (lane 2), *BamHI* (lane 3), *EcoRV* (lane 4), and *EcoRI* (lane 5) respectively, separated on a 0.8% agarose gel, and hybridized under high-stringency conditions.

To detect *W36* copy number, wheat genomic DNA was firstly digested by *Alu*I, *Sau3A*I, *Bam*HI, *Eco*RV, and *Eco*RI, respectively. Then, Southern blotting was conducted with a probe of 3'-terminus labeled by α-^32^P-dCTP. The results revealed that all DNA band were single (Figure 2). Subsequently, *W36* might be a single-copy gene in wheat genome.

### 2.3. W36 Expression Characteristics under Various Stress Conditions

**Figure 3:**
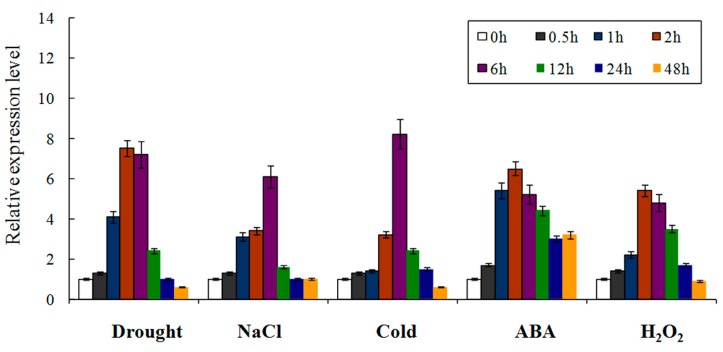
Expression of *W36* under treatments of ABA, drought, salt, H_2_O_2_, and cold. The expression of *W36* under different stimuli was normalized to the expression of wheat β*-actin*. Means and SD were calculated from three independent experiments.

The quantitative real-time PCR (qRT-PCR) results suggested that *W36* expressed differently under various stress conditions (Figure 3). Under drought treatment, the transcript level of *W36* increased within 0.5 h, reached its maximum at 2 h, and declined after 6 h. Following NaCl treatment, the expression level reached to the highest tip at 6 h and then reduced rapidly. Responding to low-temperature treatment, the expression level firstly enhanced gradually, reached rapidly to the top at 6 h, and then quickly reduced. Responding to ABA treatments, the expression level gradually increased and reached to the top at 2 h, and then gradually decreased. Treatment of H_2_O_2_ resulted in a steady state increase in the *W36* transcript reaching a peak at 2 h, which gradually came down to the basal level at 24 h. Therefore, *W36* might be regulated by high salt, drought, low-temperature, ABA, and H_2_O_2_ stresses.

### 2.4. W36 Subcellular Localization

**Figure 4:**
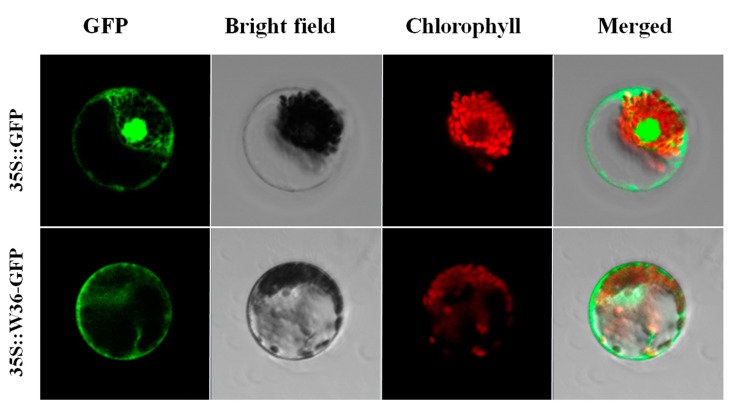
*W36* subcellular localization. The 35S::*W36*-GFP and 35S::GFP control vectors were transiently expressed in wheat protoplast cells by PEG. Results were visualized by confocal microscopy. Bars = 10 μm.

The *W36* cDNA sequence was fused to the *C*-terminus of the hGFP reporter gene and subcloned into an expression vector under the control of the CaMV 35S promoter. This construct was transferred into wheat protoplast cells by PEG (Polyethylene glycol) to investigate intra-cellular localization. Relative to the hGFP reporter, the *W36*::hGFP fusion protein was observed to be mainly localized in cytoplasm (Figure 4).

### 2.5. Characterization of the 5'-Flanking Region of W36

A 1799 bp fragment of the 5'-flanking region of the *W36* gene was obtained using I-PCR and nested PCR. To examine the putative *cis*-acting elements that may be involved in gene regulation in the 5'-flanking region, a homology search was performed using PlantCARE analysis [23]. Several potential *cis*-acting regulatory elements responsive to abiotic stresses were observed in the *W36* promoter (Table 1): (1) a heat-shock element (HSE) involved in heat stress response, and a salicylic acid response element (TCA-element) involved in salicylic acid response; (2) two ABREs involved in ABA and drought inducibility; (3) three CGTCA-motif involved in MeJA response; and (4) four EREs involved in ethylene response.

**Table 1:** Analysis of putative *cis*-acting elements in the wheat *W36* promoter.

Elements	Core Sequence	Functions
TATA-box	TAATA	Core promoter
ABRE	ACGTG	ABA and drought responsive elements
ARE	TGGTTT	Anaerobic responsive element
CGTCA-motif	CGTCA	MeJA responsive elements
ERE	ATTTCAAA	Ethylene responsive elements
TGA-element	AACGAC	Auxin responsive elements
GARE-motif	AAACAGA	Gibberellin responsive elements
GC-motif	CCCCCG	Anoxic responsive elements
HSE	AAAAAATTTC	Heat shock responsive element
TC-rich repeats	GTTTTCTTAC	Element involved in defense and stress responsiveness
TCA-element	CCATCTTTTT	Salicylic acid responsive elements

### 2.6. Overexpression of W36 Improves Tolerance to Osmotic Stress in Transgenic Plants

**Figure 5:**
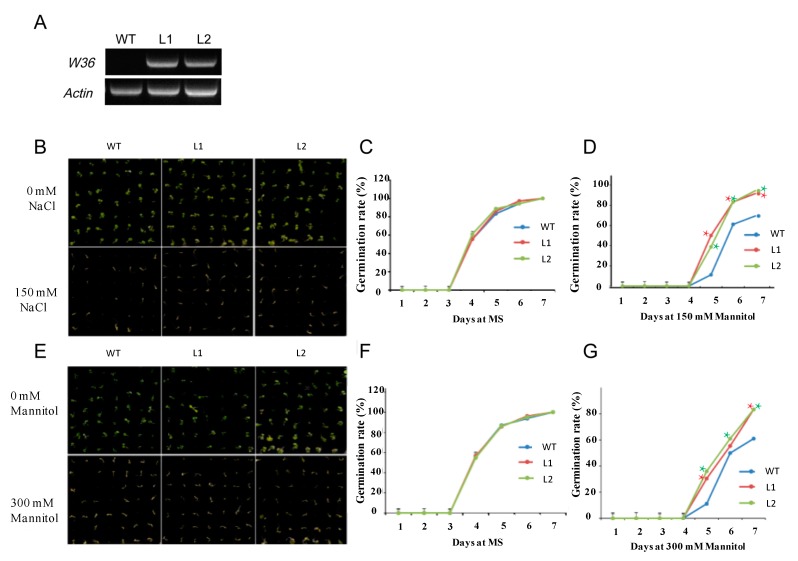
Germination of wld-type and transgenic *Arabidopsis* lines under salt and mannitol treatment. All seeds were vernalized at 4 °C for 72 h. (**A**) Expression of *W36* in transgenic lines by semi-quantitative RT-PCR. The *Actin* gene was used as a reference; (**B**,**E**) showed the seeds germination of wild-type and transgenic *Arabidopsis* lines; (**C**,**D**,**F**,**G**) showed the tendency of changing on seeds germination. Data are means ± SD of three independent experiments. Asterisks indicate significant differences (* *p* < 0.05; Student’s *t*-test).

To investigate whether *W36* could improve abiotic stress tolerance, we tested the high salinity, mannitol, drought, and cold tolerance of *W36* overexpressing plant lines (Figure 5).

Salt-tolerant testing was performed on transgenic *Arabidopsis* lines and the wild-type control. As shown in Figure 5b,c, transgenic plants and control plants cultured on MS agar medium without salt did not differ significantly in terms of seed germination. In contrast, when cultured on medium with salt (Figure 5b,d), the control showed an obvious inhibition of seed germination and growth, while the transgenic plants had a comparatively higher germination rate and better growth. Under normal culture conditions, transgenic lines and the wild-type plants had similar growth characters, with both being green and healthy. These results indicated that overexpression of *W36* was able to significantly improve the salt tolerance of transgenic *Arabidopsis*.

Additionally, we determined whether *W36* could confer increased osmotic tolerance in the transgenic *Arabidopsis*. Firstly, the ability of transgenic lines to germinate on MS medium containing 300 mM mannitol was analyzed. When germinated and grown on MS medium, *W36* transgenic plants grew almost the same as the wild type (Figure 5e). When cultured on MS medium with 300 mM mannitol for several days, however, compared with the WT line, two transgenic lines showed a significantly higher germination rate under osmotic stress (Figure 5e,f), for example, transgenic lines showed 83% germination rate, while the WT line had a germination rate of 61% (Figure 5g).

In addition, transgenic plants and control plants did not differ significantly in terms of survival rates and phenotypic changes under drought and cold treatments although *W36* responds to drought and cold (data not shown).

## 3. Experimental Section

### 3.1. Plant Materials and Stress Treatments

Wheat (*T. aestivum* cv. Xiao Baimai) seedlings were grew on seedbed at 25 °C under a 16 h light/8 h dark photoperiod for 10 days and then subjected to various stresses. Seedlings were placed in a 4 °C chamber for cold stress, and exposed to air on filter paper under 60% humidity conditions for rapid drought treatment. To mimic salinity, H_2_O_2_ and ABA treatments, seedlings were transferred into solutions containing 2% NaCl, 10 mM H_2_O_2_ and 100 µM ABA, respectively. Materials were collected at 0.5, 1, 2, 6, 12, 24, and 48 h after stress treatment.

### 3.2. De Novo Transcriptome Sequencing of Wheat

To investigate the transcriptome response to stress in wheat plant, the *de novo* transcriptome sequencing was used to perform high throughput sequence analysis. From the transcriptome sequencing data, unigenes of control and salt treatment samples collected. The genes expressing differently (FDR ≤ 0.001 and |log2Ratio| ≥ 1) in the two samples were investigated.

### 3.3. Homology Analysis

Make multiple sequences comparison and homologous analysis by GenBank and DNAMAN software. Multiple alignments of the amino acid sequences were performed using ClustalX. Phylogenetic tree was constructed using MEGA 5.1 with neighbor-joining method.

### 3.4. DNA Extraction and Southern Blotting

Wheat (*T. aestivum* cv. Xiao Baimai) genome DNA (25–30 µg) was extracted according to SDS-phenol-chloroform method, digested individually with *Alu*I, *Sau3A*I, *Bam*HI, *Eco*RV or *Eco*RI at 37 °C for one night, fractionated on 0.8% agarose gel, and then blotted onto a nylon memebrane. Southern blotting was completed using 3'-termi of *W36* labeled with α-^32^P-dCTP as a probe, washed with 2 × SSC/0.1% SDS at 65 °C, and then X-ray-plate was performed.

### 3.5. qRT-PCR Analysis

Total RNAs of the control and the treated seedlings were extracted with Trizol (TianGen, Beijing, China) kit following the manufacturer’s instructions and then treated with DNAase. First-strand cDNA was produced using 1 μg of total RNA as a template with 100 U of M-MLV reverse transcriptase (Promega, Madison, WI, USA) at 42 °C for 90 min with 1 μL Olig(dT)_15_ primer, 0.5 μL RNase inhibitor, 4 μL 5× AMV buffer, 10 mM of each dNTP in 20 μL reaction mixtures. The qRT-PCR amplifications were carried out in total reaction volumes of 20 μL, containing 10 μL 2× TransTaq HiFi PCR SuperMix (TransGen), 5 μL of the 1:5 diluted cDNA, 0.5 μL each of 20 µM forward and reverse primers and 4 μL of PCR-grade water, in an ABI7300 real-time PCR machine (Applied Biosystems, Foster City, CA, USA). The PCR program consisted of 60 °C for 45 s, 94 °C for 3 min, followed by 40 cycles of 94 °C for 15 s, and 68 °C for 31 s. *W36*RTF: 5'-GGAAAGAGAAGCAGGTTGATGTTC-3', *W36*RTR: 5'-TCATAGAAACAGCTGTACCATCACC-3' Wheat actin gene was used as an internal standard [24,25].

### 3.6. W36 Subcellular Localization

For generation of the GFP fusion protein, the coding sequences of *W36* were cloned into the *Sal*I and *Xba*I sites of hGFP. The blank GFP vector (hGFP) was used as the control. The recombinant *W36*-hGFP fusion plasmids were transformed to wheat by the PEG-mediated method [26]. Expression of fusion proteins was cultured about 12 h in darkness, and images were captured under a laser scanning confocal microscope [27].

### 3.7. Amplification of the 5'-Flanking Region of W36

To amplify the 5'-flanking region, the genomic DNA was completely digested by restriction endonuclease *Taq*I, purified using ethanol precipitation, ligated using T4 DNA ligase and then used as template. Based on the cloned sequence obtained above, the 5'-flanking region of *W36* was obtained through inverse PCR. PLACE [28], a database of plant *cis*-acting regulatory DNA elements were used to analyze the 5'-flanking region.

### 3.8. Arabidopsis Transformation and Stress Treatment

To construct an expression vector for *Arabidopsis*, the full-length *W36* cDNA was ligated into the modified vector pBI121 under the control of the CaMV35S promoter. Columbia (Col-0) ecotype *Arabidopsis* plants were transformed using the *Agrobacterium*-mediated method [29]. Transformants were selected on MS medium containing 50 μg·mL^−1^ kanamycin. T3 generation plants were used for further analysis.

In one set of experiments, seeds of wild-type and the transgenic *Arabidopsis* were placed on MS agar medium containing 150 mM NaCl and 300 mM mannitol for certain durations, respectively. Seed germination and growth were under long-day conditions (15 h light/9 h dark) at 22 °C. The percentage of germinated seeds was calculated based on the number of seedlings that reached the cotyledon stage at 2 weeks [26].

For drought treatment, plants were grown at normal growing conditions without watering for 2 weeks; and then rewatered under normal growing conditions. Cold treatments were performed by transferring the plants in a pre-cooled medium to growth chambers at −6 °C for 3 h, and then rewatered under normal growing conditions. Survival rates were assessed after two weeks [30].

## 4. Discussion

Plants possess sophisticated signaling networks that can perceive and respond to hormones and environmental stresses. V-type H^+^-ATPase conducted proton motive force (PMF) and pumped H^+^ from cytoplasma into vacuole using the energy produced by hydrolyzing cytoplasma ATP. Thereby, V-type H^+^-ATPase could motive solute transportation between cytoplasma and vacuole, maintain solute relatively constant, and guarantee normal life activities. In this present study, the wheat *W36* mainly localized in cytoplasm (Figure 4). Many studies take evidences for the core status of V-type H^+^-ATPase on mataining the cytoplasma pH, Ca^2+^ concentration, water potential, metabolites steady-state, and stress responses [31,32,33,34,35]. Therefore, *W36* maybe functions in cytoplasm under stress conditions.

Under salt stress conditions, the cell survival is strongly dependent on adaptively maintaining or adjusting the activity of the V-type H^+^-ATPase [36]. Under high salt condition, the hydrolyzing and enzyme activity of V-H^+^-ATPase were obviously increased to promote transcription of A, B, H, and C subunits and expression products accumulation [8]. Binzel *et al.* [37] found that expression of V-type H^+^-ATPase respond to salt stress was not only related with the treatment time but also tissue specificity. After treated on salt condition for 24 h, mRNA of V-type H^+^-ATPase A subunit of tomato was increased and recovered to normal condition after seven days. While in the young leaf and root, there were both not obvious changes. V-type H^+^-ATPase E subunit had weak response to salt stress [38]. All above revealed that salt stress affected V-type H^+^-ATPase expression differently in glycophytes and halophytes.

In order to discern the possible modulation mechanism of V-type H^+^-ATPase activity of wheat under salt stress, the expression of *W36* was analyzed. The results in Figure 3 indicated that the transcript of *W36* was obviously up-regulated by NaCl stresses. Overexpression of the *W36* by transgenic *Arabidopsis* was able to enhance seed germination and adult seedling growth under salt stress. Batelli *et al.* [39] reported that regulation of V-type H^+^-ATPase activity is an additional key function of SOS2 in coordinating changes in ion transport during salt stress and in promoting salt tolerance. Salt stress could lead to an increase in ABA content, and ABA participates in the SOS signal pathway through regulating an increase in Ca^2+^ concentration [40]. High cytosolic Ca^2+^ level can induce the phosphorylation of different proteins in cells [41]. Evidence has also been presented that in the V-type H^+^-ATPase phosphorylation, as a target of activation by NaCl, a calcium/calmodulin-dependent protein kinase sensitive to staurosporin is involved [42,43]. Some reports displayed that exogenous ABA could notably promote the activity of V-type H^+^-ATPase [34,44,45]. In this paper, ABA treatment also caused an increase of the transcript level of *W36*. These results indicate that overexpressing *W36* could increase V-type H^+^-ATPase activity, thereby providing more energy for ion transporters to decrease the cellular Na^+^ toxicity to enhance plant salt tolerance.

ABA, one of the “classical” plant hormones, regulates many aspects of plant growth and development, including seed dormancy and germination [46,47,48]. H_2_O_2_ as a signaling molecule plays a significant role in the response to the stress caused by reactive oxygen species (ROS) in plant. Intersection of ABA and H_2_O_2_ signaling has been documented in previous studies. Evidence for ABA in triggering the production of H_2_O_2_ [49] and H_2_O_2_ up-regulating the ABA catabolism genes [50] has been provided. In addition, studies of ABA action demonstrate that ABA-mediated regulation of stomatal closure requires H_2_O_2_ [51]. Considering the complexity of plant signaling networks, more information will be needed to study the subunits coordinate and act together on improving holoenzyme activity, which could be important to discern the real role of *W36* contributed to V-type H^+^-ATPase in response to environmental stimuli.

## 5. Conclusions

*W36*, a single-copy gene, encoded the E subunit of the V-type H^+^-ATPase that accumulated in the cytoplasm in wheat. qRT-PCR analysis revealed that *W36* could be induced by drought, cold, salt, and exogenous ABA treatment, which corresponds to the containment of stress-responsive elements in *W36* promoter. Overexpression of *W36* enhanced salt and mannitol tolerance in transgenic *Arabidopsis* plants. Therefore, *W36* is involved in the plant response to osmotic stress.
